# Processed and Unprocessed Red Meat and Risk of Colorectal Cancer: Analysis by Tumor Location and Modification by Time

**DOI:** 10.1371/journal.pone.0135959

**Published:** 2015-08-25

**Authors:** Adam M. Bernstein, Mingyang Song, Xuehong Zhang, An Pan, Molin Wang, Charles S. Fuchs, Ngoan Le, Andrew T. Chan, Walter C. Willett, Shuji Ogino, Edward L. Giovannucci, Kana Wu

**Affiliations:** 1 Rally Health, San Francisco, California, United States of America; 2 Department of Nutrition, Harvard T. H. Chan School of Public Health, Boston, Massachusetts, United States of America; 3 Department of Epidemiology, Harvard T. H. Chan School of Public Health, Boston, Massachusetts, United States of America; 4 Department of Epidemiology and Biostatistics, School of Public Health, Tongji Medical College, Huazhong University of Science and Technology, Hubei, China; 5 Department of Biostatistics, Harvard T. H. Chan School of Public Health, Boston, Massachusetts, United States of America; 6 Department of Medical Oncology, Dana-Farber Cancer Institute and Harvard Medical School, Boston, Massachusetts, United States of America; 7 Hanoi Medical University, Hanoi, Vietnam; 8 Channing Division of Network Medicine, Department of Medicine, Brigham and Women’s Hospital and Harvard Medical School, Boston, Massachusetts, United States of America; 9 Division of Gastroenterology, Massachusetts General Hospital, Boston, Massachusetts, United States of America; 10 Department of Pathology, Brigham and Women’s Hospital and Harvard Medical School, Boston, Massachusetts, United States of America; Tufts University, UNITED STATES

## Abstract

Although the association between red meat consumption and colorectal cancer (CRC) is well established, the association across subsites of the colon and rectum remains uncertain, as does time of consumption in relation to cancer development. As these relationships are key for understanding the pathogenesis of CRC, they were examined in two large cohorts with repeated dietary measures over time, the Nurses’ Health Study (n = 87,108 women, 1980–2010) and Health Professionals Follow-up Study (n = 47,389 men, 1986–2010). Cox proportional hazards regression models generated hazard ratios (HRs) and 95% confidence intervals (CIs), which were pooled by random-effects meta-analysis. In combined cohorts, there were 2,731 CRC cases (1,151 proximal colon, 816 distal colon, and 589 rectum). In pooled analyses, processed red meat was positively associated with CRC risk (per 1 serving/day increase: HR = 1.15, 95% CI: 1.01–1.32; *P* for trend 0.03) and particularly with distal colon cancer (per 1 serving/day increase; HR = 1.36; 95% CI: 1.09–1.69; *P* for trend 0.006). Recent consumption of processed meat (within the past 4 years) was not associated with distal cancer. Unprocessed red meat was inversely associated with risk of distal colon cancer and a weak non-significant positive association between unprocessed red meat and proximal cancer was observed (per 1 serving/day increase: distal HR = 0.75; 95% CI: 0.68–0.82; *P* for trend <0.001; proximal HR = 1.14, 95% CI: 0.92–1.40; *P* for trend 0.22). Thus, in these two large cohorts of US health professionals, processed meat intake was positively associated with risk of CRC, particularly distal cancer, with little evidence that higher intake of unprocessed red meat substantially increased risk of CRC. Future studies, particularly those with sufficient sample size to assess associations by subsites across the colon are needed to confirm these findings and elucidate potentially distinct mechanisms underlying the relationship between processed meat and subtypes of unprocessed red meat with CRC.

## Introduction

As colorectal cancer (CRC) is the third most common cancer worldwide following lung and breast cancer, and the fourth most common cause of cancer death after lung, stomach, and liver cancer, identifying strategies to reduce its incidence is paramount [1]. Red and processed meat consumption have been associated with an increased risk of CRC in many observational studies and thus reducing, or eliminating, their intake may prevent CRC development [2–5]. Yet the impact of these meats on CRC risk may depend on the time in one’s life when meat is consumed [6–9] and meat consumption may impact cancer development differently across the three regions of the colorectum (proximal colon, distal colon, or rectum) [10–15]. To date, however, evidence on the relation between red meat and CRC risk by subsite location and time of intake has been limited. Understanding the temporal relation between meat consumption and CRC development and meat consumption in relation to cancer development in CRC regions are key for understanding CRC pathogenesis.

We previously reported that in two large cohorts of US men and women, intake of red meat, and particularly that from beef, lamb and pork as a main dish, as well as of processed meat, was positively associated with risk of colon cancer, but not of rectal cancer [14, 16–18]. In order to better understand the association between unprocessed and processed red meat and CRC risk by tumor subsite location and time of red meat intake, we returned to our two prospective cohorts of U.S. men and women.

## Materials and Methods

### Study Populations

The Nurses’ Health Study (NHS) began in 1976 when 121,700 U.S. female registered nurses aged 30–55 provided information on their medical history and lifestyle. The Health Professionals Follow-up Study (HFPS) began in 1986 when 51,529 U.S. male dentists, pharmacists, optometrists, osteopaths, podiatrists, and veterinarians aged 40–75 years provided information on their medical history and lifestyle. Every two years, follow-up questionnaires have been sent to update both cohorts’ information. In 1980, a validated 61-item food-frequency questionnaire (FFQ) was included to assess intake of specific foods in the NHS. Expanded FFQs updated dietary intake in 1984 and every four years between 1986 and 2010. In 1986, a similar validated 131-item FFQ was used in the HPFS and administered every four years between 1990 and 2010. As in our previous analyses, we excluded participants with excessive blank items on the baseline FFQ (≥10 of the 61 FFQ items in 1980 for women or ≥70 on the 131-item FFQ for men) or implausibly low or high energy intake (<600 or >3500 kcal/day for women and <800 or >4,200 kcal/day for men), and those with previously diagnosed cancer (except non-melanoma skin cancer) or ulcerative colitis [16]. The final baseline populations (in 1980 for NHS and 1986 for HPFS) consisted of 87,108 women and 47,389 men.

### Ascertainment of Diet

To calculate intakes of unprocessed and processed red meat, a commonly used unit or portion size for each food was specified on the FFQ and the participant was asked how often on average during the previous year he or she had consumed that amount. Answers ranged from “never” to “more than six times per day.” Nutrient intake was calculated by multiplying the frequency by the nutrient composition in a standard portion size of that food and then summing the nutrient intake from all food items. The food composition database was created primarily from U.S.D.A. sources [19]. For unprocessed red meat consumption, the FFQ included questions on “beef or lamb as main dish,” “pork as main dish,” “hamburger,” and “beef, pork, or lamb as a sandwich or mixed dish.” For processed red meat, there were questions on “bacon,” “beef or pork hot dogs,” “salami, bologna, or other processed meat sandwiches,” and “other processed red meats such as sausage, kielbasa, etc.” The reproducibility and validity of the FFQs in measuring food intake have been previously described [20–22].

### Ascertainment of Cancer

For both cohorts, each mailed questionnaire asked whether a participant was diagnosed with CRC or any other disease within the previous two years. When a participant reported a diagnosis of CRC, we contacted the participants for permission to review medical records. Study physicians reviewed the medical records to confirm a CRC diagnosis and determine histological type, anatomic location, and cancer stage. Colorectal cancer was classified according to the International Classification of Diseases, Ninth Revision [ICD-9] [23, 24]. Proximal cancers included those that occurred in the cecum, ascending colon, and transverse colon (ICD-9 codes 153.0, 153.1, 153.4, 153.6, 153.7) while distal cancers were those located in the descending and sigmoid colon (ICD-9 codes 153.2 and 153.3). Rectosigmoid cancers were grouped with rectal cancers (ICD-9 codes 154.0 or 154.1). We used information from next-of kin, state vital statistics records, the National Death Index, and the postal system to identify deaths in the cohorts [25]. Cause of death was determined after review of death certificates and medical records.

### Data Analysis

Each participant contributed person-time of follow-up from the date of return of the baseline questionnaire to the date of CRC diagnosis, death, loss to follow-up, or end of analysis follow-up (June 1, 2010 for NHS and January 31, 2010 for HPFS), whichever came first. We used Cox proportional hazards regression models to calculate the hazard ratios (HRs) and 95% confidence intervals (CIs) for the association between unprocessed and processed red meat and risk of CRC and its subsites. To control as finely as possible for confounding by age, calendar time, and a possible interactions between these two time scales, we stratified models jointly by age (in months) and 2-year questionnaire cycle.

We grouped unprocessed and processed red meat intake into categories of servings/day. Continuous measures of red meat intake generated the *P*-for-trend across categories and were used to estimate the HRs and 95% CIs for a 1-serving-per-day increase in intake. We examined potential non-linear relations with restricted cubic splines and the *P* for non-linearity was examined by a likelihood ratio test comparing the model with the linear term to the model with the linear and cubic spline terms [26]. None of the *P* values for non-linearity reached statistical significance.

In multivariable models, we adjusted for potential dietary and non-dietary confounders, including body mass index, pack-years of smoking before age 30, first-degree relatives with a history of CRC, history of endoscopy, menopausal status (in women), current aspirin or non-steroidal anti-inflammatory drug (NSAID) use, alcohol intake, folate, calcium, vitamin D, and energy intake, and physical exercise. Details on covariates are found in the footnotes to results tables. In sensitivity analyses, we mutually adjusted for unprocessed and processed red meat and then, separately, for other meats (fish and poultry), the Alternative Healthy Eating Index (AHEI) score [27], Dietary Approaches to Stop Hypertension (DASH) score [28], fruit and vegetable intake, dietary cholesterol, saturated fat, and animal protein intake. We also evaluated for effect modification of the main results by BMI (≤ 25 or > 25 kg/m^2^), physical activity (< or ≥ 17 MET-hrs/wk in NHS or < or ≥ 35 MET-hrs/wk in HPFS), family history of CRC (yes/no), or smoking history (never/ever).

To evaluate possible modification by time of the association between unprocessed and processed red meat intake and risk of CRC, the following models were constructed. The cumulative average intake model, widely used in prior studies to minimize the impact of random error in reporting of dietary intake and to best reflect long-term habits, was considered the primary model [29].

#### Model 1: Cumulative average

In this model, unprocessed and processed red meat represents the cumulative average intake since 1980 (NHS) or 1986 (HPFS), calculated as the mean of unprocessed or processed red meat from all available FFQs until the beginning of each follow-up interval.

#### Model 2: Simple update (0–4 year lag)

In this model, meat intake represents the most recently reported intake; that is, the intake reported on the most recent FFQ before each follow-up interval.

#### Model 3: Baseline only

In this model, meat intake values are derived directly from the 1980 (NHS) and 1986 (HPFS) questionnaires.

#### Models 4–6: Latency analyses

In these models, we examined associations between unprocessed and processed red meat intake and CRC incidence at different latencies after dietary assessment by associating unprocessed and processed red meat with CRC events 4–8, 8–12, and 12–16 years after reported intake. For example, in the 8–12 year lagged analysis, we evaluated the relationship between unprocessed and processed red meat intake in 1986 with the risk of CRC between 1994 and 1998. If a participant had missing dietary information for a particular FFQ cycle, he or she was excluded from the analysis.

We pooled results from the NHS and HPFS and generated summary estimates using random-effects models [30]. To assess heterogeneity of the associations between red meat and cancer subsite for each cohort, we used Cox proportional cause-specific hazards regression models with a duplication method for competing risks data [31]. For the pooled analyses, we tested whether a risk factor had a statistically different regression coefficient for different tumor subsites using a 3-degree-of-freedom X^2^ test based on the contrast statistic (one degree for each CRC subsite) [32]. All reported *P* values are 2-sided with <0.05 considered statistically significant.

### Ethics Statement

The study was approved by the Committee on the use of Human Subjects in Research at Brigham and Women’s Hospital and the Institutional Review Board of the Harvard T. H. Chan School of Public Health, both in Boston. Completion and return of the questionnaire was considered to imply informed consent.

## Results

### Participant Characteristics

We documented 1,735 CRCs (809 proximal colon, 514 distal colon, and 373 rectal cancers) in women during 2,439,732 person-years of follow-up in the NHS and 996 CRCs (342 proximal colon, 303 distal colon, and 216 rectal cancers) in men during 1,013,022 person-years of follow-up in the HPFS. Characteristics of study participants, averaged according to proportion of person-time in each category of intake, are shown in Table 1. Men and women with higher red meat consumption tended to have a higher BMI, lower physical activity, higher intake of alcohol, and lower intakes of fish, folate, calcium, and vitamin D compared to participants with lower red meat intake.

**Table 1:** Age-standardized characteristics of person-years according to frequency of total red meat intake in the Nurses’ Health Study and in the Health Professionals Follow-up Study^a^.

Characteristic	Nurses’ Health Study			Health Professionals Follow-up Study		
	0 to ≤3 svg/wk	>5 svg/wk to ≤1 svg/d	>2 svg/d	0 to ≤3 svg/wk	>5 svg/wk to ≤1 svg/d	>2 svg/d
**Person-years**	632,152	375,160	67,100	237,166	141,194	48,650
**Total red meat intake, g/d**	25.5 ± 14.4	78.5 ± 20.9	210 ± 73.1	21.3 ± 13.5	73.3 ± 16.6	197 ± 60.9
**Age, yrs**	59.6 ± 10.9	58.9 ± 10.8	58.6 ± 10.9	62.6 ± 11.0	62.5 ± 11.0	62.3 ± 11.0
**Body mass index, kg/m** ^**2**^ ^**b**^	25.2 ± 4.7	26.2 ± 5.2	27.1 ± 6.0	24.9 ± 3.8	25.8 ± 4.2	26.7 ± 4.8
**Physical activity, MET-hours/wk** ^**c**^	19.8 ± 24.7	15.8 ± 20.8	13.7 ± 20.9	36.8 ± 41.2	33.6 ± 39.8	34.9 ± 44.4
**Pack-years of smoking before age 30**	7.0 ± 5.5	7.0 ± 5.3	7.2 ± 5.3	10.6 ± 6.6	11.0 ± 6.5	12.1 ± 7.0
**Colorectal cancer in a parent or sibling, %**	16.8	17.1	17.7	14.6	14.0	13.9
**History of previous endoscopy, %**	21.2	19.5	17.0	28.2	26.0	21.9
**Current multivitamin use, %**	56.3	50.4	45.3	54.3	49.9	44.3
**Regular aspirin or NSAID use, %** ^**d**^	47.4	50.7	52.6	49.3	50.7	50.7
**Postmenopausal, %**	74.0	71.8	70.4	-	-	-
**Current postmenopausal hormone use, %** ^**e**^	52.5	49.0	43.3	-	-	-
**Dietary intake**						
** Unprocessed red meat, servings/d**	0.21 ± 0.13	0.60 ± 0.19	1.47 ± 0.62	0.18 ± 0.12	0.58 ± 0.19	1.45 ± 0.62
** Processed red meat, servings/d**	0.06 ± 0.07	0.26 ± 0.17	1.03 ± 0.67	0.05 ± 0.07	0.27 ± 0.18	1.16 ± 0.79
** Poultry, servings/d**	0.37 ± 0.31	0.36 ± 0.27	0.43 ± 0.44	0.43 ± 0.37	0.41 ± 0.32	0.48 ± 0.48
** Fish, servings/d**	0.27 ± 0.27	0.24 ± 0.21	0.22 ± 0.23	0.37 ± 0.34	0.29 ± 0.25	0.27 ± 0.26
** Total fruit, servings/d**	2.44 ± 1.50	2.28 ± 1.37	2.26 ± 1.47	2.95 ± 1.82	2.49 ± 1.55	2.42 ± 1.63
** Total vegetable, servings/d**	3.01 ± 1.78	2.99 ± 1.62	3.28 ± 1.87	3.46 ± 2.01	3.28 ± 1.76	3.78 ± 2.05
** Alcohol, g/d**	5.12 ± 9.22	6.10 ± 10.6	6.13 ± 11.4	9.11 ± 12.8	12.2 ± 15.5	13.4 ± 17.7
** Animal protein, g/d**	49.2 ± 17.4	53.1 ± 13.5	63.1 ± 14.6	56.6 ± 20.4	61.9 ± 15.9	72.9 ± 16.5
** Vegetable protein, g/d**	22.8 ± 6.2	19.9 ± 4.8	16.5 ± 4.9	30.9 ± 8.1	26.4 ± 5.7	22.8 ± 5.2
** Folate, μg/d**	478 ± 303	393 ± 217	323 ± 170	718 ± 396	613 ± 331	524 ± 280
** Calcium, mg/d**	1279 ± 640	1062 ± 524	837 ± 433	1096 ± 543	974 ± 440	848 ± 361
** Vitamin D, IU/d**	482 ± 342	390 ± 279	315 ± 233	534 ± 354	440 ± 284	373 ± 240
** Dietary fiber, g/d**	20.6 ± 6.7	17.2 ± 5.1	14.3 ± 4.8	26.9 ± 8.6	21.9 ± 6.2	18.7 ± 5.3
** Total fat, g/d**	50.2 ± 12.5	59.2 ± 11.2	70.5 ± 12.7	59.7 ± 15.0	70.9 ± 12.7	81.7 ± 13.0
** Saturated fat, g/d**	16.7 ± 5.6	20.9 ± 5.5	26.0 ± 6.4	18.1 ± 5.8	23.4 ± 5.3	28.1 ± 5.6
** Heme iron, mg/d**	0.74 ± 0.37	1.07 ± 0.40	1.64 ± 0.62	0.89 ± 0.43	1.21 ± 0.43	1.67 ± 0.51
** Cholesterol, mg/d**	204 ± 103	245 ± 89.5	313 ± 110	218 ± 99.2	273 ± 95.2	351 ± 125.6
** DASH score**	26.4 ± 4.4	22.9 ± 4.3	20.5 ± 4.3	27.0 ± 5.0	23.2 ± 4.8	21.2 ± 4.7
** AHEI score**	52.0 ± 9.90	44.3 ± 9.0	38.5 ± 9.2	48.1 ± 10.6	40.3 ± 9.2	37.3 ± 8.6

^a^ Lowest, intermediate, and highest category of 5 categories shown; Continuous variables are described as mean ± standard deviation; DASH = Dietary Approaches to Stop Hypertension; AHEI = Alternate Healthy Eating Index.

^b^ Body mass index is calculated as weight in kilograms divided by height in meters squared.

^c^ Physical activity is represented by the product sum of the metabolic equivalent (MET) of each specific recreational activity and hours spent on that activity per week.

^d^ Regular users are defined as ≥2 standard (325-mg) tablets of aspirin or ≥ 2 tablets of non-steroidal anti-inflammatory drugs (NSAIDs) per week.

^e^ Proportion of current postmenopausal hormone use is calculated among postmenopausal women only.

### Results for Risk of Colorectal Cancer and its Subsites Among Men and Women Combined

Pooled, age-adjusted results showed a modest significant positive association between processed red meat and CRC and a weak positive association for unprocessed red meat and CRC (Table 2). After multivariable adjustment, the processed red meat association was attenuated (cumulative average HR for 1-serving-per-day (HR), 95% confidence interval (CI): 1.15, 1.01–1.32) and that for unprocessed red meat became null (cumulative average HR for 1-serving-per-day: 0.99, 0.87–1.13). *(From here on*, *HRs and 95% CIs for 1-serving-per-day increments are reported for cumulative average intake unless otherwise noted*.*)*

**Table 2:** Pooled hazard ratio of colorectal cancer according to 1-serving-per-day increase of red meat intake by time of exposure^a^.

	Baseline	Simple update (0-4-year lag)	4-8-year lag	8-12-year lag	12-16-year lag	Cumulative average
**No. of cases**	2,731	2,336	2,036	1,701	1,403	2,731
**Person-years**	3,452,754	2,830,020	2,451,734	2,029,538	1,592,112	3,452,754
**Total red meat**						
** Age-adjusted HR (95% CI)**	1.10 (1.04–1.15)	1.12 (1.05–1.20)	1.14 (1.06–1.23)	1.14 (1.00–1.29)	1.14 (1.03–1.25)	1.16 (1.08–1.24)
***P* for trend**	0.0005	<0.001	<0.001	0.05	0.01	<0.001
** Multivariable-adjusted HR (95% CI)**	1.04 (0.98–1.11)	1.02 (0.94–1.11)	1.09 (0.99–1.19)	1.04 (0.86–1.27)	1.05 (0.88–1.26)	1.06 (0.97–1.16)
***P* for trend**	0.19	0.59	0.07	0.66	0.58	0.19
**Unprocessed red meat**						
** Age-adjusted HR (95% CI)**	1.09 (1.01–1.18)	1.16 (1.05–1.29)	1.17 (1.04–1.31)	1.14 (0.99–1.31)	1.13 (0.99–1.30)	1.15 (1.03–1.28)
***P* for trend**	0.03	0.004	0.008	0.07	0.07	0.01
** Multivariable-adjusted HR (95% CI)**	1.02 (0.94–1.12)	1.04 (0.92–1.17)	1.09 (0.96–1.25)	1.01 (0.87–1.17)	1.02 (0.87–1.18)	0.99 (0.87–1.13)
***P* for trend**	0.61	0.52	0.18	0.87	0.84	0.88
**Processed red meat**						
** Age-adjusted HR (95% CI)**	1.17 (1.07–1.27)	1.18 (1.05–1.33)	1.23 (1.08–1.40)	1.26 (1.09–1.47)	1.24 (0.98–1.57)	1.31 (1.17–1.46)
***P* for trend**	<0.001	0.006	0.001	0.002	0.07	<0.001
** Multivariable-adjusted HR (95% CI)**	1.08 (0.98–1.18)	1.02 (0.89–1.17)	1.13 (0.98–1.31)	1.10 (0.84–1.43)	1.11 (0.74–1.67)	1.15 (1.01–1.32)
***P* for trend**	0.13	0.74	0.09	0.50	0.60	0.03

^a^ Each multivariable model adjusted for age, 2-year follow-up cycle, family history of colorectal cancer, prior lower gastrointestinal endoscopy, pack-years of smoking before age 30 (0, 0–4, 4–10, >10), body mass index (in kg/m^2^; <22, 22–24, 24–25, 25–27, 27–29, 29–30, 30–32, 32–35, 35–40, or ≥40), physical activity (in metabolic equivalent-hours/week; <3, 3–9, 9–18, 18–27, or ≥27), current multivitamin use, postmenopausal status and hormone use in women (premenopausal, and never, past and current users of postmenopausal hormone), regular aspirin or NSAID use (≥2 tablets/week), total caloric intake (quintiles), alcohol consumption (in g/d; <5, 5–10, 10–15, 15–30, or ≥30), and energy-adjusted intake of folate (quintiles), calcium (quintiles), vitamin D (quintiles) and total fiber (quintiles). Continuous measures of red meat intake generated the P-for-trend across categories and were used to estimate the HRs and 95% CIs for a 1-serving-per-day increase in intake.

When risk estimates were pooled for each subsite, in age-adjusted models higher consumption of unprocessed red meat was associated with a higher risk of proximal colon cancer (HR, 95% CI: 1.25, 1.06–1.47) but the association was attenuated and became non-significant after multivariable adjustment (HR, 95% CI: 1.14, 0.92–1.40) (Table 3). Unprocessed red meat was not associated with distal colon cancer in age-adjusted models but after multivariable adjustment a significant inverse association was observed with distal colon cancer (HR, 95% CI: 0.75, 0.68–0.82; *P* for heterogeneity (proximal vs. distal) = 0.06). Higher processed red meat intake was significantly associated with higher risk of distal colon cancer in both age-adjusted and multivariable-adjusted models (multivariable HR, 95% CI: 1.36, 1.09–1.69), but not with proximal colon cancers (multivariable HR, 95% CI: 0.99, 0.79–1.24; *P* for heterogeneity proximal vs. distal = 0.07). Associations between cumulatively updated unprocessed and processed red meat and risk of rectal cancer were positive in age-adjusted models but were attenuated after multivariable adjustment and did not reach statistical significance [multivariable HR per 1 serving/day for unprocessed red meat: 1.14 (95% CI: 0.86, 1.51) with *P* for trend 0.09; for processed red meat: 1.18 (0.89, 1.57) with *P* for trend 0.25]. We did not find evidence that observed associations for unprocessed or processed red meat differed between colon and rectal cancers (all *P* for heterogeneity for colon vs. rectum ≥ 0.30 in NHS and ≥ 0.74 in HPFS).

**Table 3:** Pooled hazard ratio of colorectal cancer subsites according to 1-serving-per-day increase of red meat intake by cancer subsites and time of exposure^a^.

	Baseline	Simple update (0-4-year lag)	4-8-year lag	8-12-year lag	12-16-year lag	Cumulativeaverage
**Proximal colon cancer**						
**Cases**	1,151	1,007	898	784	647	1,151
**Total red meat**						
** Age-adjusted HR (95% CI)**	1.11 (1.02–1.20)	1.10 (0.99–1.23)	1.12 (0.99–1.25)	1.06 (0.90–1.26)	1.09 (0.87–1.37)	1.15 (1.04–1.28)
***P* for trend**	0.01	0.07	0.06	0.46	0.43	0.009
** Multivariable-adjusted HR (95% CI)**	1.05 (0.96–1.16)	1.00 (0.88–1.14)	1.09 (1.00–1.19)	1.02 (0.90–1.16)	1.04 (0.88–1.22)	1.06 (0.92–1.22)
***P* for trend**	0.27	0.95	0.06	0.74	0.68	0.44
**Unprocessed red meat**						
** Age-adjusted HR (95% CI)**	1.18 (1.04–1.35)	1.19 (1.01–1.40)	1.18 (0.99–1.41)	1.13 (0.83–1.54)	1.11 (0.88–1.39)	1.25 (1.06–1.47)
***P* for trend**	0.01	0.04	0.06	0.43	0.38	0.008
** Multivariable-adjusted HR (95% CI)**	1.13 (0.99–1.29)	1.07 (0.89–1.29)	1.14 (1.00–1.30)	1.08 (0.88–1.31)	1.03 (0.89–1.20)	1.14 (0.92–1.40)
***P* for trend**	0.07	0.48	0.05	0.47	0.66	0.22
**Processed red meat**						
** Age-adjusted HR (95% CI)**	1.08 (0.94–1.24)	1.11 (0.91–1.34)	1.16 (0.94–1.43)	1.07 (0.83–1.37)	1.13 (0.77–1.65)	1.17 (0.97–1.41)
***P* for trend**	0.27	0.30	0.18	0.59	0.55	0.11
** Multivariable-adjusted HR (95% CI)**	0.98 (0.84–1.15)	0.95 (0.76–1.18)	1.10 (0.94–1.29)	1.00 (0.83–1.20)	1.04 (0.78–1.40)	0.99 (0.79–1.24)
***P* for trend**	0.82	0.62	0.23	0.97	0.78	0.93
**Distal colon cancer**						
**Cases**	817	696	594	473	382	817
**Total red meat**						
** Age-adjusted HR (95% CI)**	1.11 (1.01–1.21)	1.10 (0.97–1.24)	1.12 (0.98–1.29)	1.20 (1.03–1.40)	1.17 (0.86–1.59)	1.15 (1.02–1.30)
***P* for trend**	0.04	0.14	0.09	0.02	0.32	0.02
** Multivariable-adjusted HR (95% CI)**	1.04 (0.93–1.16)	0.95 (0.82–1.11)	1.10 (0.99–1.22)	1.14 (1.02–1.28)	1.14 (0.93–1.39)	1.01 (0.86–1.19)
***P* for trend**	0.52	0.51	0.08	0.03	0.21	0.86
**Unprocessed red meat**						
** Age-adjusted HR (95% CI)**	1.00 (0.86–1.15)	1.02 (0.84–1.24)	1.02 (0.82–1.27)	1.10 (0.86–1.40)	1.04 (0.58–1.85)	0.99 (0.81–1.20)
***P* for trend**	0.97	0.84	0.86	0.46	0.91	0.88
** Multivariable-adjusted HR (95% CI)**	0.88 (0.75–1.05)	0.82 (0.66–1.03)	0.97 (0.82–1.15)	1.00 (0.84–1.21)	0.97 (0.67–1.41)	0.75 (0.68–0.82)
***P* for trend**	0.16	0.10	0.75	0.94	0.88	<0.001
**Processed red meat**						
** Age-adjusted HR (95% CI)**	1.31 (1.14–1.51)	1.28 (1.04–1.56)	1.35 (1.09–1.68)	1.50 (1.20–1.89)	1.46 (1.12–1.90)	1.50 (1.25–1.80)
***P* for trend**	<0.001	0.02	0.006	<0.001	0.005	<0.001
** Multivariable-adjusted HR (95% CI)**	1.23 (1.05–1.44)	1.09 (0.86–1.38)	1.31 (1.12–1.54)	1.43 (1.20–1.70)	1.42 (1.16–1.74)	1.36 (1.09–1.69)
***P* for trend**	0.009	0.48	0.001	<0.001	<0.001	0.006
**Rectal cancer**						
**Cases**	589	509	441	362	306	589
**Total red meat**						
** Age-adjusted HR (95% CI)**	1.08 (0.96–1.21)	1.17 (1.01–1.35)	1.19 (1.02–1.38)	1.21 (1.02–1.44)	1.13 (0.93–1.38)	1.19 (1.03–1.37)
***P* for trend**	0.20	0.03	0.03	0.03	0.23	0.02
** Multivariable-adjusted HR (95% CI)**	1.04 (0.90–1.20)	1.14 (0.96–1.35)	1.19 (1.06–1.34)	1.20 (1.05–1.37)	1.11 (0.93–1.31)	1.14 (0.94–1.37)
***P* for trend**	0.57	0.13	0.003	0.008	0.25	0.18
**Unprocessed red meat**						
** Age-adjusted HR (95% CI)**	1.08 (0.91–1.28)	1.24 (0.96–1.61)	1.27 (1.01–1.59)	1.24 (0.95–1.61)	1.11 (0.83–1.48)	1.21 (0.97–1.52)
***P* for trend**	0.38	0.10	0.04	0.12	0.49	0.09
** Multivariable-adjusted HR (95% CI)**	1.05 (0.84–1.32)	1.22 (0.95–1.58)	1.29 (1.08–1.53)	1.22 (1.00–1.49)	1.07 (0.86–1.34)	1.14 (0.86–1.51)
***P* for trend**	0.66	0.12	0.005	0.05	0.52	0.37
**Processed red meat**						
** Age-adjusted HR (95% CI)**	1.13 (0.93–1.36)	1.24 (0.97–1.58)	1.27 (0.98–1.65)	1.35 (0.89–2.05)	1.24 (0.83–1.85)	1.33 (1.04–1.70)
***P* for trend**	0.22	0.09	0.08	0.16	0.29	0.02
** Multivariable-adjusted HR (95% CI)**	1.05 (0.85–1.30)	1.14 (0.86–1.50)	1.25 (1.03–1.53)	1.31 (0.94–1.83)	1.19 (0.88–1.63)	1.18 (0.89–1.57)
***P* for trend**	0.64	0.35	0.03	0.10	0.26	0.25

^a^ Each multivariable model adjusted for age, 2-year follow-up cycle, family history of colorectal cancer, prior lower gastrointestinal endoscopy, pack-years of smoking before age 30 (0, 0–4, 4–10, >10), body mass index (in kg/m^2^; <22, 22–24, 24–25, 25–27, 27–29, 29–30, 30–32, 32–35, 35–40, or ≥40), physical activity (in metabolic equivalent-hours/week; <3, 3–9, 9–18, 18–27, or ≥27), current multivitamin use, postmenopausal status and hormone use in women (premenopausal, and never, past and current users of postmenopausal hormone), regular aspirin or NSAID use (≥2 tablets/week), total caloric intake (quintiles), alcohol consumption (in g/d; <5, 5–10, 10–15, 15–30, or ≥30), and energy-adjusted intake of folate (quintiles), calcium (quintiles), vitamin D (quintiles) and total fiber. (quintiles). Continuous measures of red meat intake generated the P-for-trend across categories and were used to estimate the HRs and 95% CIs for a 1-serving-per-day increase in intake.

### Assessment of Potential Time Modification

We did not observe evidence for consistent or meaningful effect modification by time between unprocessed red meat intake and risk of CRC, or cancer in the proximal colon, distal colon, or rectum (Tables 2 and 3). Our results suggest, however, a higher risk of distal colon cancer after a lag of at least 4–8 years from time of consumption of processed red meat (0–4 year lag: 1.09, 95% CI: 0.86–1.38; 4–8 year lag: 1.31, 95% CI: 1.12–1.54; 8–12 year lag: 1.43, 95% CI: 1.20–1.70; 12–16 year lag: 1.42, 95% CI: 1.16–1.74). Furthermore, we observed statistically significant positive associations between unprocessed and processed red meat and rectal cancer after a 4–8 year lag: multivariable HR for unprocessed meat with 4–8 year lag: 1.29 (95% CI: 1.08, 1.53) with *P* for trend: 0.005; multivariable HR for processed meat with 4–8 year lag: 1.25 (95% CI: 1.03, 1.53) with *P* for trend: 0.03).

### Sex-Specific Analyses

Analyses conducted among men and women separately and prior to pooling are presented in tables in S1, S2, S3 and S4 Files. Associations between processed and unprocessed red meat and risk of CRC, proximal colon, distal colon, and rectal cancers were largely similar in direction and magnitude to those observed in pooled analyses with few exceptions. For the cumulative average intake of processed red meat and distal colon cancer, the HR was 1.37 (95% CI, 1.02–1.85, with *P* for trend of 0.04) in women and 1.35 (95% CI, 0.98–1.85, with *P* for trend of 0.07) in men.

### Estimates by Gram Increments

To compare our results with earlier studies and account for variation in serving size across different types of red meat (e.g. a serving of a hot dog versus that of bacon), we examined CRC and subsite associations per 50g/day increment for processed red meat and 100g and 120g/day for unprocessed red meat [5]. Results were similar to those observed for per-serving-day increments (Table in S5 File).

### Sensitivity Analyses

Results remained largely unchanged for CRC risk after separate adjustment for other meats (fish and poultry), the Alternative Healthy Eating Index (AHEI) score, Dietary Approaches to Stop Hypertension (DASH) score, dietary cholesterol, saturated fat, and animal protein intake, as well as after mutual adjustment for unprocessed or processed red meat (data not shown). The hazard ratio of distal colon cancer associated with 1 serving/day increment in processed red meat intake was 1.35 (95% CI, 1.09–1.68, P for trend = 0.007) after adjusting for fruits and vegetable intake. Among men, after adjusting for animal protein, the HR for distal colon cancer for cumulative average intake of unprocessed red meat was 0.79 (95% CI: 0.53–1.18) per 1 serving-per-day increment, and after adjusting for vegetable protein the HR was 0.72 (95% CI: 0.49–1.07). Among women, after adjusting for animal protein, the HR of distal colon cancer for cumulative average intake of unprocessed red meat was 0.73 (0.50–1.04) per 1 serving-per-day increment, and after adjusting for vegetable protein the HR was 0.80 (95% CI: 0.56–1.12). In pooled analyses, after adjusting for total protein, the HR was 0.74 (95% CI: 0.57–0.96).

In additional sensitivity analyses, there was no significant difference in our main findings after including poultry hot dogs in the processed meat category or after excluding smokers at baseline. With adjustment for processed red meat, fish, and poultry, the HR for a 1 serving-per-day increase of unprocessed red meat was 1.13 (95% CI: 0.91–1.40) for proximal colon cancer and 0.72 (95% CI: 0.56–0.92) for distal colon cancer. For processed red meat, the HR for proximal colon cancer was 0.94 (95% CI: 0.75–1.19) and for distal colon cancer, it was 1.43 (95% CI: 1.15–1.78).

To examine whether changes in questions after the NHS 1980 FFQ may have affected the association between processed red meat intake and distal cancer risk, we undertook an analysis by starting follow-up in NHS in 1986. Results were largely unchanged: a high intake of processed red meat was associated with an increased risk of distal colon cancer (HR: 1.41; 95% CI: 0.98–2.02 per 1 serving/day increment, with *P* for trend = 0.06).

### Assessment of Potential Confounding and Effect Modification

To assess which covariate(s) accounted most for the observed differences between age-adjusted and multivariable estimates (Tables 2 and 3), we constructed a “base model” with total caloric intake and standard non-dietary risk factors for CRC (age, family history of CRC, endoscopy, pack-years of smoking before age 30, BMI, physical activity, and aspirin/NSAID use) and then separately added one dietary covariate at a time to the model. In women, calcium intake, and in men, fiber, folate, and calcium intake accounted most for the observed changes in risk estimates between unprocessed red meat and distal colon cancers. In both cohorts, positive associations between processed red meat and distal colon cancers were attenuated but remained relatively stable upon adjusting for dietary covariates.

In men, physical activity and family history appeared to modify the association between unprocessed red meat and proximal colon cancer: among men with low physical activity, the HR per 1-serving-per day increase was 1.41 (95% CI: 0.98–2.03) and for those with high activity it was 0.78 (95 CI: 0.45–1.38), with a *P* for interaction of 0.06. Among men with a family history of CRC the HR per 1-serving-per day increase was 1.76 (95% CI: 1.09–2.84) and for those without a family history it was 1.00 (95 CI: 0.68–1.46), with a *P* for interaction of 0.04. Among women, family history also appeared to modify associations, but only for distal colon cancer. Among women with a family history of CRC, the HR per 1-serving-per day increase in unprocessed red meat was 1.12 (95 CI: 0.66–1.89) and for those without a family history, it was 0.63 (95 CI: 0.44–0.91), with a *P* for interaction of 0.05. For all other stratified analyses, *P* for interaction ≥ 0.06. Notably, there were few cases with a positive family history (92 proximal colon cancers in men and 112 distal colon cancers in women).

## Discussion

In these two large cohorts of men and women with repeated measures of dietary intake and over two decades of follow-up, we found a positive association between processed red meat intake and distal colon cancer and an inverse association between unprocessed red meat intake and distal colon cancer. There was a weak, statistically non-significant, positive association between unprocessed red meat and risk of proximal colon cancer. Recent processed meat intake within the past four years was not associated with risk of distal colon cancer.

Together, these findings build on a large body of research on the relation between red and processed meat intake and CRC risk. In 2007, the World Cancer Research Fund/American Institute for Cancer Research (WCRF/AICR) stated there was convincing evidence linking high red and processed meat consumption with an increased risk of CRC [33] and subsequent systematic reviews and meta-analyses, which included studies published after the WCRF/AICR report, provided further evidence for a positive association [5, 34]. In studies that have examined unprocessed (fresh) red and processed meats separately, associations between processed meat and CRC were generally stronger (at least two fold) and more consistent across studies [2, 3, 5, 35, 36]. For instance, although the 2007 WCRF/AICR and the 2011 Continuous Update Project both concluded that the evidence for red and processed meat and CRC is convincing, in the 2007 report, 100g/day of red meat intake increased risk of CRC by 29%, while in the 2011 report that estimate dropped to 17%. Observed estimates for processed meat, on the other hand, were fairly stable (per 50g/day increase: 21% in 2007 and 18% in 2011) [33, 37].

Earlier case-control studies examined associations between red and processed meat and CRC by subsite but, for the most part, focused on colon versus rectal cancers, and led to inconsistent results (reviewed by Norat *et al* in 2001 and 2002 [4, 36]). In a recent large population-based case-control study by Miller *et al*., no significant associations were identified between unprocessed red meat and CRC, regardless of tumor location [38]. In that study, higher processed meat intake was specifically and significantly associated with an increased risk of proximal colon cancer.

Only a limited number of prospective studies have looked at the relation between red and processed meat and development of CRC by subsite across the colon (i.e. proximal vs. distal colon). A meta-analysis of three cohorts by Larsson *et al* published one year prior to the WCRF/AICR report observed that associations for red meat tend to be stronger for rectal cancers, whereas processed meat associations were stronger for distal cancers [2]. Consistent with the meta-analysis by Larsson *et al*., a meta-analysis performed by Chan *et al*. [5] that incorporated prospective studies not included in the 2007 WCRF/AICR report found that positive associations for processed meat were stronger for distal than proximal cancers, although the test for heterogeneity was not statistically significant and results were based on only two cohort studies [11, 13]; it also did not include two large cohorts, the European Investigation into Cancer and Nutrition (EPIC) cohort and the NIH-AARP Diet and Health Study (NIH-AARP) [10, 12]. In the EPIC study, associations for processed meats appeared to be stronger for distal colon than proximal colon cancers [12], while in the NIH-AARP study, positive associations did not differ by colon subsite but did appear to be stronger for rectal cancers [10]. In an updated analysis from the Multiethnic Cohort Study, published about one year after the meta-analysis by Chan *et al*. [5], red and processed meat intake was not associated with risk of CRC or any of the three CRC subsites [39].

Another recent prospective study conducted within the Norwegian Women Cancer cohort study found that higher processed meat intake, but not unprocessed red meat, was associated with higher risk of CRC in all three subsites [40].

Despite decades of research, clear mechanisms underlying the association between red meat and CRC remain unknown. The proximal colon, distal colon, and rectum arise from different embryonic tissues, serve different biologic functions, and are exposed to fecal matter for different durations of time [41]. Carcinogenesis across the regions of the colon and rectum may thus stem from different molecular pathways. Proximal colon cancers are more likely than rectal and distal colon tumors to have microsatellite instability, a CpG island methylator phenotype, and *KRAS* mutations, whereas rectal and distal colon tumors are more likely than proximal colon tumors to have *TP53 and APC* mutations [42]. In addition, carcinogenesis in various regions of the colon and rectum may also be impacted differently by exposure to processed and unprocessed meat. Processed meat contains additives such as nitrates and nitrites, which are precursors to *N*-nitroso compounds (NOC) that may act as alkylating agents and generate DNA damage such as G >A transitions [43–47]. Heme, but not non-heme, iron, in red meat can induce endogenous production of NOCs, which may at least, in part, contribute to the observed positive associations between total red meat and unprocessed red meat and CRC [48, 49]. Interestingly, data suggest that associations between red meat and CRC may also differ by subtype of red meat investigated. For example, in a recent meta-analysis, higher intake of beef was associated with higher risk of colon, but not rectal, cancer and higher intake of lamb was associated with higher risk of CRC; on the other hand, pork, which contains a lower amount of heme iron than beef or lamb, was not associated with risk of CRC [50]. However, in that study moderate-to-high study heterogeneity was noted with pork intake and summary estimates were based on a limited number of studies (beef: 5 studies; pork: 5 studies; lamb: 2 studies). Therefore, more studies are needed to confirm these findings. Both unprocessed and processed red meat cooked well done at high temperatures are also a source of meat mutagens, such as heterocyclic amines (HCA) which are known carcinogens in animal models [10]. Findings from the NIH–AARP study indicated that NOCs and HCAs may differentially impact risk of cancer across the colorectum [10]. While such mechanisms may help explain our findings of a positive association between processed red meat intake and distal colon cancer, human studies on dietary intake of nitrate, nitrite, NOCs and HCA and risk of CRC have been inconsistent, and prospective data remain limited [10, 39, 51–53].

We observed that higher unprocessed red meat intake was associated with a lower risk of distal colon cancer but that associations were only observed after adjusting for calcium, folate, and fiber intake. These findings are contrary to those reported by the NIH-AARP and EPIC studies and the Swedish Mammography Cohort study [10–12]; however, a recent case-control study did find an inverse association between unprocessed red meat intake and distal colon cancer among whites but not African-Americans [54]. In the case control study, adjustment for total protein intake attenuated the inverse association, suggesting that protein rather than red meat *per se* may at least, in part, drive the inverse association. Adjustment for protein intake did not materially change our pooled results and, even though inverse associations were primarily seen in our study after adjustment for specific nutrients associated with a healthier diet, we cannot exclude the possibility of residual confounding to explain differences between our results and those of prior studies. Notably, too, we found some evidence suggesting that associations between unprocessed red meat and proximal or distal cancers may differ by family history of CRC; however, the number of cases with a positive family history was small (92 proximal colon cancers in men and 112 distal colon cancers in women) and we cannot exclude the possibility of a chance finding.

As colorectal cancer likely develops over decades, diet may have a changing impact on cancer development over the life course [6–9]. Studies suggest that early energy restriction during childhood [7–9] and higher consumption of vegetables and vitamin A during adolescence [6] decrease the risk of CRC in middle age. Compared to participants with low red or processed meat intake in both adolescence and adulthood, high red and processed meat in either adolescence or adulthood was associated with increased risk of colon cancer, and associations appear to be strongest for those with high red or processed meat intake at both time points [6]. In addition, long-term intake of red meat, rather than acute (most recent) intake, has been associated with a higher risk of CRC [13]. Our results suggest a higher risk between processed red meat intake and distal colon cancer after a latency period of at least 4–8 years. These findings point to a need to also examine dietary exposures in earlier stages of life.

The strengths of our study include its prospective design, long follow-up, high follow-up rate, and large sample size. Detailed and repeated measures of unprocessed and processed red meat intake and of established risk factors for CRC allowed us to conduct latency and sensitivity analyses not available in previous studies. Although measurement of dietary data is imperfect, repeated measurements serve to reduce random error, and random error that occurs would likely lead to an underestimation of the true association between the exposures and outcome. A limitation of this study is that we did not examine associations between meat mutagens produced during the cooking process (e.g. heterocyclic amines) or the doneness of meat and risk of CRC. Many diet and lifestyle behaviors are associated with red meat consumption and we carefully adjusted for potential confounders; nevertheless we cannot exclude the possibility of residual or unmeasured confounding due to the observational nature of this study.

In conclusion, in these two large cohorts of US health professionals, we found little evidence that higher intake of unprocessed red meat substantially increased risk of CRC. On the other hand, we observed a significant and positive association between processed red meat and CRC, particularly distal colon cancer. Recent processed meat intake was not associated with risk of distal colon cancer. Further studies, particularly those with sufficient sample size to examine associations separately by subsites across the colon, are needed to confirm these findings and elucidate potentially distinct pathways underlying the relationship between processed meat and subtypes of unprocessed red meat and CRC.

## Supporting Information

S1 File Hazard ratios and 95% confidence intervals of colorectal cancer in Nurses’ Health Study. (DOCX)

S2 File Hazard ratios and 95% confidence intervals of colorectal cancer in Health Professionals Follow-up Study. (DOCX)

S3 File Hazard ratios and 95% confidence intervals of colorectal cancer by subsite in Nurses’ Health Study. (DOCX)

S4 File Hazard ratios and 95% confidence intervals of colorectal cancer by subsite in Health Professionals Follow-up Study. (DOCX)

S5 File Hazard ratios and 95% confidence intervals with intake of 100 and 120 g/day of red meat and 30 and 50 g/day of processed meats by cancer subsite. (DOCX)
