# Supplementary material for: Processed and Unprocessed Red Meat and Risk of Colorectal Cancer: Analysis by Tumor Location and Modification by Time
Source: PLoS One. 2015 Aug 25;10(8):e0135959. doi: 10.1371/journal.pone.0135959 (PMC4549221; doi:10.1371/journal.pone.0135959)
Supplement: S1 File — (DOCX) [file pone.0135959.s001.docx]

**S1 Table. Hazard ratios (HRs) and 95% confidence intervals (95% CIs) of colorectal cancer according to red meat intake among 87,108 women in the Nurses’ Health Study ^a^**

|  | **Baseline** | **Simple update**  **(0-4-year lag)** | **4-8-year lag** | **8-12-year lag** | **12-16-year lag** | **Cumulative average** |
| --- | --- | --- | --- | --- | --- | --- |
| **No. of cases/person-years** | 1,735/2,439,732 | 1,479/2,026,460 | 1,341/1,768,524 | 1,178/1,486,648 | 1,039/1,190,356 | 1,735/2,439,732 |
| **Total red meat** |  |  |  |  |  |  |
| **0 to ≤3 svg/wk** | 1.00 (ref) | 1.00 (ref) | 1.00 (ref) | 1.00 (ref) | 1.00 (ref) | 1.00 (ref) |
| **>3 to ≤5 svg/wk** | 0.99 (0.82-1.18) | 1.05 (0.91-1.21) | 1.03 (0.89-1.19) | 0.98 (0.84-1.14) | 0.99 (0.84-1.16) | 0.99 (0.83-1.16) |
| **>5 svg/wk to ≤1 svg/d** | 1.11 (0.93-1.32) | 1.19 (1.02-1.39) | 1.20 (1.02-1.41) | 1.03 (0.86-1.22) | 1.03 (0.85-1.24) | 1.06 (0.89-1.26) |
| **>1 to ≤2 svg/d** | 1.02 (0.86-1.21) | 1.07 (0.91-1.27) | 1.13 (0.95-1.34) | 0.97 (0.80-1.17) | 0.95 (0.77-1.16) | 1.00 (0.83-1.21) |
| **>2 svg/d** | 1.19 (0.95-1.49) | 1.16 (0.82-1.64) | 1.06 (0.71-1.60) | 1.11 (0.73-1.68) | 1.10 (0.67-1.80) | 1.01 (0.71-1.42) |
| ***P* for trend** | 0.56 | 0.45 | 0.51 | 0.45 | 0.62 | 0.43 |
| **HR (95% CI) for 1-serving-per-day increase** | 1.02 (0.95-1.11) | 1.04 (0.93-1.17) | 1.04 (0.92-1.18) | 0.95 (0.83-1.09) | 0.96 (0.83-1.12) | 1.05 (0.93-1.19) |
| **Unprocessed red meat** |  |  |  |  |  |  |
| **0 to ≤2 svg/wk** | 1.00 (ref) | 1.00 (ref) | 1.00 (ref) | 1.00 (ref) | 1.00 (ref) | 1.00 (ref) |
| **>2 to ≤3 svg/wk** | 0.99 (0.83-1.17) | 0.98 (0.85-1.14) | 1.03 (0.88-1.20) | 0.90 (0.76-1.05) | 0.93 (0.78-1.11) | 0.90 (0.76-1.07) |
| **>3 to ≤5 svg/wk** | 1.10 (0.93-1.30) | 1.11 (0.96-1.29) | 1.14 (0.98-1.33) | 1.02 (0.87-1.20) | 1.01 (0.85-1.20) | 1.01 (0.86-1.19) |
| **>5 svg/wk to ≤1 svg/d** | 0.96 (0.81-1.13) | 1.15 (0.96-1.37) | 1.15 (0.96-1.39) | 1.03 (0.84-1.27) | 0.90 (0.72-1.11) | 0.94 (0.78-1.14) |
| **>1 svg/d** | 1.05 (0.87-1.25) | 1.11 (0.89-1.38) | 1.11 (0.88-1.40) | 0.87 (0.67-1.14) | 0.94 (0.73-1.21) | 0.96 (0.76-1.21) |
| ***P* for trend** | 0.90 | 0.26 | 0.41 | 0.66 | 0.93 | 0.78 |
| **HR (95% CI) for 1-serving-per-day increase** | 1.01 (0.90-1.12) | 1.10 (0.94-1.28) | 1.07 (0.91-1.27) | 0.96 (0.79-1.16) | 0.99 (0.82-1.20) | 0.98 (0.81-1.17) |
| **Processed red meat** |  |  |  |  |  |  |
| **0 svg/wk** | 1.00 (ref) | 1.00 (ref) | 1.00 (ref) | 1.00 (ref) | 1.00 (ref) | 1.00 (ref) |
| **0.1 to 0.5 svg/wk** | 1.07 (0.87-1.32) | 1.07 (0.91-1.27) | 1.02 (0.86-1.22) | 1.01 (0.84-1.22) | 1.00 (0.83-1.21) | 1.30 (0.96-1.75) |
| **>0.5 svg/wk to ≤1 svg/wk** | 1.06 (0.87-1.28) | 1.02 (0.85-1.21) | 1.00 (0.84-1.20) | 1.05 (0.87-1.26) | 1.02 (0.84-1.25) | 1.23 (0.91-1.66) |
| **>1 to ≤3 svg/wk** | 1.03 (0.86-1.22) | 1.12 (0.96-1.31) | 1.17 (1.00-1.38) | 1.06 (0.89-1.26) | 1.05 (0.87-1.26) | 1.31 (0.99-1.74) |
| **>3 svg/wk** | 1.08 (0.90-1.30) | 1.03 (0.85-1.24) | 1.07 (0.88-1.31) | 1.01 (0.81-1.25) | 0.89 (0.70-1.13) | 1.28 (0.95-1.73) |
| ***P* for trend** | 0.41 | 0.77 | 0.66 | 0.73 | 0.46 | 0.10 |
| **HR (95% CI) for 1-serving-per-day increase** | 1.06 (0.93-1.20) | 1.03 (0.85-1.25) | 1.05 (0.85-1.30) | 0.96 (0.76-1.22) | 0.91 (0.70-1.18) | 1.17 (0.97-1.42) |

^a^ Cox proportional hazards model adjusted for age, 2-year follow-up cycle, family history of colorectal cancer, prior lower gastrointestinal endoscopy, pack-years of smoking before age 30 (0, 0-4, 4-10, >10), body mass index (in kg/m^2^; <22, 22-24, 24-25, 25-27, 27-29, 29-30, 30-32, 32-35, 35-40, or ≥40), physical activity (in metabolic equivalent-hours/week; <3, 3-9, 9-18, 18-27, or ≥27), current multivitamin use, postmenopausal status and hormone use (premenopausal, and never, past and current users of postmenopausal hormone), regular aspirin or NSAID use (≥2 tablets/week), total caloric intake (quintiles), alcohol consumption (in g/d; <5, 5-10, 10-15, 15-30, or ≥30), and energy-adjusted intake of folate (quintiles), calcium (quintiles), vitamin D (quintiles) and total fiber (quintiles).
