# Supplementary material for: Processed and Unprocessed Red Meat and Risk of Colorectal Cancer: Analysis by Tumor Location and Modification by Time
Source: PLoS One. 2015 Aug 25;10(8):e0135959. doi: 10.1371/journal.pone.0135959 (PMC4549221; doi:10.1371/journal.pone.0135959)
Supplement: S2 File — (DOCX) [file pone.0135959.s002.docx]

**S2 Table. Hazard ratios (HRs) and 95% confidence intervals (95% CIs) of colorectal cancer according to red meat intake among 47,389 men in the Health Professionals Follow-up Study ^a^**

|  | **Baseline** | **Simple update**  **(0-4-year lag)** | **4-8-year lag** | **8-12-year lag** | **12-16-year lag** | **Cumulative average** |
| --- | --- | --- | --- | --- | --- | --- |
| **No. of cases/person-years** | 996/1,013,022 | 857/803,560 | 695/684,364 | 523/544,128 | 364/402,466 | 996/1,013,022 |
| **Total red meat** |  |  |  |  |  |  |
| **0 to ≤3 svg/wk** | 1.00 (ref) | 1.00 (ref) | 1.00 (ref) | 1.00 (ref) | 1.00 (ref) | 1.00 (ref) |
| **>3 to ≤5 svg/wk** | 0.93 (0.76-1.14) | 0.86 (0.69-1.06) | 0.85 (0.67-1.08) | 0.85 (0.64-1.11) | 1.33 (0.97-1.81) | 1.10 (0.90-1.34) |
| **>5 svg/wk to ≤1 svg/d** | 1.02 (0.83-1.26) | 0.99 (0.80-1.22) | 1.05 (0.83-1.34) | 1.26 (0.96-1.64) | 1.15 (0.82-1.62) | 1.11 (0.89-1.37) |
| **>1 to ≤2 svg/d** | 1.11 (0.92-1.34) | 1.05 (0.86-1.29) | 1.18 (0.94-1.48) | 1.15 (0.88-1.51) | 1.31 (0.96-1.80) | 1.25 (1.01-1.55) |
| **>2 svg/d** | 1.22 (0.91-1.63) | 0.90 (0.64-1.28) | 1.22 (0.83-1.78) | 1.21 (0.78-1.88) | 1.47 (0.90-2.43) | 1.20 (0.85-1.70) |
| ***P* for trend** | 0.17 | 0.98 | 0.04 | 0.05 | 0.09 | 0.28 |
| **HR (95% CI) for 1-serving-per-day increase** | 1.07 (0.97-1.18) | 1.00 (0.89-1.12) | 1.14 (1.01-1.30) | 1.16 (1.00-1.34) | 1.16 (0.98-1.37) | 1.07 (0.95-1.22) |
| **Unprocessed red meat** |  |  |  |  |  |  |
| **0 to ≤2 svg/wk** | 1.00 (ref) | 1.00 (ref) | 1.00 (ref) | 1.00 (ref) | 1.00 (ref) | 1.00 (ref) |
| **>2 to ≤3 svg/wk** | 0.97 (0.80-1.17) | 0.95 (0.77-1.16) | 1.04 (0.82-1.31) | 0.97 (0.75-1.27) | 1.25 (0.92-1.71) | 1.02 (0.83-1.25) |
| **>3 to ≤5 svg/wk** | 0.89 (0.73-1.09) | 1.12 (0.92-1.36) | 1.21 (0.97-1.51) | 1.23 (0.96-1.58) | 1.03 (0.75-1.41) | 1.05 (0.87-1.27) |
| **>5 svg/wk to ≤1 svg/d** | 1.00 (0.83-1.21) | 0.99 (0.79-1.24) | 1.10 (0.86-1.43) | 1.06 (0.79-1.43) | 1.07 (0.75-1.52) | 1.04 (0.84-1.30) |
| **>1 svg/d** | 0.99 (0.78-1.26) | 0.95 (0.73-1.23) | 1.14 (0.86-1.51) | 1.09 (0.79-1.51) | 1.23 (0.86-1.78) | 0.98 (0.75-1.27) |
| ***P* for trend** | 0.46 | 0.73 | 0.26 | 0.41 | 0.67 | 0.93 |
| **HR (95% CI) for 1-serving-per-day increase** | 1.06 (0.91-1.24) | 0.97 (0.80-1.17) | 1.12 (0.92-1.37) | 1.10 (0.87-1.38) | 1.06 (0.82-1.38) | 1.01 (0.83-1.23) |
| **Processed red meat** |  |  |  |  |  |  |
| **0 svg/wk** | 1.00 (ref) | 1.00 (ref) | 1.00 (ref) | 1.00 (ref) | 1.00 (ref) | 1.00 (ref) |
| **0.1 to ≤0.5 svg/wk** | 1.24 (0.96-1.60) | 1.13 (0.89-1.45) | 1.16 (0.87-1.52) | 1.03 (0.76-1.40) | 0.96 (0.65-1.41) | 1.25 (0.93-1.68) |
| **>0.5 svg/wk to ≤1 svg/wk** | 1.15 (0.89-1.48) | 0.95 (0.74-1.23) | 1.15 (0.86-1.51) | 0.86 (0.62-1.19) | 1.38 (0.96-2.00) | 1.23 (0.91-1.66) |
| **>1 to ≤3 svg/wk** | 1.21 (0.96-1.51) | 1.11 (0.89-1.38) | 1.23 (0.96-1.58) | 1.08 (0.84-1.44) | 1.50 (1.07-2.08) | 1.40 (1.07-1.84) |
| **>3 svg/wk** | 1.37 (1.09-1.73) | 1.17 (0.92-1.48) | 1.45 (1.11-1.89) | 1.38 (1.02-1.86) | 1.61 (1.12-2.30) | 1.62 (1.21-2.16) |
| ***P* for trend** | 0.18 | 0.85 | 0.06 | 0.07 | 0.02 | 0.17 |
| **HR (95% CI) for 1-serving-per-day increase** | 1.10 (0.96-1.27) | 1.02 (0.84-1.23) | 1.21 (0.99-1.47) | 1.26 (0.99-1.61) | 1.37 (1.06-1.78) | 1.14 (0.95-1.37) |

^a^ Cox proportional hazards model adjusted for age, 2-year follow-up cycle, family history of colorectal cancer, prior lower gastrointestinal endoscopy, pack-years of smoking before age 30 (0, 0-4, 4-10, >10), body mass index (in kg/m^2^; <22, 22-24, 24-25, 25-27, 27-29, 29-30, 30-32, 32-35, 35-40, or ≥40), physical activity (in metabolic equivalent-hours/week; <3, 3-9, 9-18, 18-27, or ≥27), current multivitamin use, regular aspirin or NSAID use (≥2 tablets/week), total caloric intake (quintiles), alcohol consumption (in g/d; <5, 5-10, 10-15, 15-30, or ≥30), and energy-adjusted intake of folate (quintiles), calcium (quintiles), vitamin D (quintiles) and total fiber (quintiles).
