# Supplementary material for: Processed and Unprocessed Red Meat and Risk of Colorectal Cancer: Analysis by Tumor Location and Modification by Time
Source: PLoS One. 2015 Aug 25;10(8):e0135959. doi: 10.1371/journal.pone.0135959 (PMC4549221; doi:10.1371/journal.pone.0135959)
Supplement: S3 File — (DOCX) [file pone.0135959.s003.docx]

**S3 Table. Hazard ratios (HRs) and 95% confidence intervals (95% CIs) of colorectal cancer according to 1-serving-per-day increase of red meat by subsite among women in the Nurses’ Health Study ^a^**

|  | **Baseline** | **Simple update**  **(0-4-year lag)** | **4-8-year lag** | **8-12-year lag** | **12-16-year lag** | **Cumulative average** |
| --- | --- | --- | --- | --- | --- | --- |
| **Proximal colon cancer** |  |  |  |  |  |  |
| **No. of cases** | 809 | 700 | 651 | 584 | 512 | 809 |
| **Total red meat** |  |  |  |  |  |  |
| **HR (95% CI)†** | 1.05 (0.94-1.18) | 1.05 (0.89-1.24) | 1.05 (0.88-1.25) | 0.87 (0.71-1.07) | 0.85 (0.68-1.05) | 1.06 (0.87-1.28) |
| ***P* for trend** | 0.36 | 0.56 | 0.58 | 0.18 | 0.13 | 0.57 |
| **Unprocessed red meat** |  |  |  |  |  |  |
| **HR (95% CI)†** | 1.11 (0.95-1.29) | 1.13 (0.90-1.43) | 1.09 (0.85-1.39) | 0.88 (0.66-1.16) | 0.89 (0.67-1.18) | 1.10 (0.84-1.44) |
| ***P* for trend** | 0.19 | 0.30 | 0.49 | 0.35 | 0.41 | 0.47 |
| **Processed red meat** |  |  |  |  |  |  |
| **HR (95% CI)†** | 0.99 (0.82-1.21) | 1.02 (0.76-1.37) | 1.07 (0.79-1.46) | 0.83 (0.57-1.19) | 0.72 (0.48-1.09) | 1.03 (0.76-1.40) |
| ***P* for trend** | 0.94 | 0.91 | 0.66 | 0.31 | 0.12 | 0.85 |
| **Distal colon cancer** |  |  |  |  |  |  |
| **No. of cases** | 514 | 430 | 375 | 319 | 280 | 514 |
| **Total red meat** |  |  |  |  |  |  |
| **HR (95% CI)†** | 1.01 (0.87-1.16) | 0.91 (0.74-1.12) | 1.00 (0.80-1.26) | 1.04 (0.82-1.34) | 1.29 (1.00-1.65) | 0.99 (0.80-1.24) |
| ***P* for trend** | 0.91 | 0.38 | 0.98 | 0.73 | 0.05 | 0.95 |
| **Unprocessed red meat** |  |  |  |  |  |  |
| **HR (95% CI)†** | 0.88 (0.72-1.09) | 0.81 (0.60-1.10) | 0.93 (0.67-1.30) | 0.90 (0.62-1.29) | 1.25 (0.90-1.75) | 0.73 (0.53-1.01) |
| ***P* for trend** | 0.24 | 0.17 | 0.66 | 0.56 | 0.18 | 0.06 |
| **Processed red meat** |  |  |  |  |  |  |
| **HR (95% CI)†** | 1.18 (0.96-1.46) | 1.05 (0.75-1.46) | 1.16 (0.81-1.67) | 1.36 (0.95-1.95) | 1.45 (0.99-2.10) | 1.37 (1.02-1.85) |
| ***P* for trend** | 0.11 | 0.78 | 0.42 | 0.09 | 0.05 | 0.04 |
| **Rectal cancer** |  |  |  |  |  |  |
| **No. of cases** | 373 | 321 | 289 | 253 | 223 | 373 |
| **Total red meat** |  |  |  |  |  |  |
| **HR (95% CI)†** | 0.98 (0.83-1.16) | 1.19 (0.95-1.50) | 1.07 (0.83-1.39) | 1.05 (0.79-1.38) | 0.88 (0.64-1.23) | 1.08 (0.83-1.41) |
| ***P* for trend** | 0.83 | 0.13 | 0.59 | 0.74 | 0.46 | 0.57 |
| **Unprocessed red meat** |  |  |  |  |  |  |
| **HR (95% CI)†** | 0.96 (0.76-1.22) | 1.36 (0.99-1.85) | 1.20 (0.84-1.71) | 1.26 (0.86-1.84) | 0.90 (0.59-1.38) | 1.03 (0.70-1.51) |
| ***P* for trend** | 0.74 | 0.05 | 0.32 | 0.23 | 0.63 | 0.88 |
| **Processed red meat** |  |  |  |  |  |  |
| **HR (95% CI)†** | 1.01 (0.76-1.33) | 1.06 (0.70-1.61) | 0.96 (0.60-1.53) | 0.85 (0.50-1.45) | 0.77 (0.42-1.39) | 1.16 (0.78-1.74) |
| ***P* for trend** | 0.97 | 0.77 | 0.86 | 0.55 | 0.38 | 0.45 |

^a^  Cox proportional hazards model adjusted for age, 2-year follow-up cycle, family history of colorectal cancer, prior lower gastrointestinal endoscopy, pack-years of smoking before age 30 (0, 0-4, 4-10, >10), body mass index (in kg/m^2^; <22, 22-24, 24-25, 25-27, 27-29, 29-30, 30-32, 32-35, 35-40, or ≥40), physical activity (in metabolic equivalent-hours/week; <3, 3-9, 9-18, 18-27, or ≥27), current multivitamin use, postmenopausal status and hormone use (premenopausal, and never, past and current users of postmenopausal hormone), regular aspirin or NSAID use (≥2 tablets/week), total caloric intake (quintiles), alcohol consumption (in g/d; <5, 5-10, 10-15, 15-30, or ≥30), and energy-adjusted intake of folate (quintiles), calcium (quintiles), vitamin D (quintiles) and total fiber (quintiles).
