# Supplementary material for: Processed and Unprocessed Red Meat and Risk of Colorectal Cancer: Analysis by Tumor Location and Modification by Time
Source: PLoS One. 2015 Aug 25;10(8):e0135959. doi: 10.1371/journal.pone.0135959 (PMC4549221; doi:10.1371/journal.pone.0135959)
Supplement: S4 File — (DOCX) [file pone.0135959.s004.docx]

**S4 Table. Hazard ratios (HRs) and 95% confidence intervals (95% CIs) of colorectal cancer according to 1-serving-per-day increase of red meat by subsite among men in the Health Professionals Follow-up Study ^a^**

|  | **Baseline** | **Simple update**  **(0-4-year lag)** | **4-8-year lag** | **8-12-year lag** | **12-16-year lag** | **Cumulative average** |
| --- | --- | --- | --- | --- | --- | --- |
| **Proximal colon cancer** |  |  |  |  |  |  |
| **No. of cases** | 342 | 307 | 247 | 200 | 135 | 342 |
| **Total red meat** |  |  |  |  |  |  |
| **HR (95% CI)†** | 1.06 (0.89-1.25) | 0.94 (0.77-1.15) | 1.05 (0.84-1.31) | 1.14 (0.88-1.47) | 1.15(0.87-1.52) | 1.06 (0.86-1.30) |
| ***P* for trend** | 0.53 | 0.56 | 0.69 | 0.32 | 0.32 | 0.60 |
| **Unprocessed red meat** |  |  |  |  |  |  |
| **HR (95% CI)†** | 1.19 (0.92-1.55) | 0.97 (0.71-1.32) | 1.09 (0.77-1.53) | 1.26 (0.88-1.82) | 1.11 (0.73-1.69) | 1.19 (0.86-1.65) |
| ***P* for trend** | 0.18 | 0.82 | 0.63 | 0.21 | 0.62 | 0.30 |
| **Processed red meat** |  |  |  |  |  |  |
| **HR (95% CI)†** | 0.96 (0.74-1.25) | 0.86 (0.62-1.20) | 0.99 (0.68-1.45) | 1.07 (0.68-1.69) | 1.30 (0.82-2.06) | 0.95 (0.68-1.32) |
| ***P* for trend** | 0.76 | 0.38 | 0.97 | 0.76 | 0.27 | 0.74 |
| **Distal colon cancer** |  |  |  |  |  |  |
| **No. of cases** | 303 | 266 | 219 | 154 | 102 | 303 |
| **Total red meat** |  |  |  |  |  |  |
| **HR (95% CI)†** | 1.09 (0.91-1.30) | 1.00 (0.80-1.24) | 1.12 (0.89-1.42) | 1.09 (0.83-1.43) | 0.85 (0.59-1.23) | 1.04 (0.82-1.31) |
| ***P* for trend** | 0.37 | 0.97 | 0.33 | 0.54 | 0.39 | 0.74 |
| **Unprocessed red meat** |  |  |  |  |  |  |
| **HR (95% CI)†** | 0.88 (0.65-1.20) | 0.84 (0.60-1.19) | 0.88 (0.59-1.32) | 0.90 (0.58-1.40) | 0.56 (0.32-0.99) | 0.75 (0.51-1.09) |
| ***P* for trend** | 0.43 | 0.33 | 0.54 | 0.64 | 0.05 | 0.13 |
| **Processed red meat** |  |  |  |  |  |  |
| **HR (95% CI)†** | 1.30 (1.03-1.65) | 1.13 (0.81-1.58) | 1.33 (0.97-1.83) | 1.28 (0.84-1.95) | 1.23 (0.71-2.10) | 1.35 (0.98-1.85) |
| ***P* for trend** | 0.03 | 0.47 | 0.08 | 0.26 | 0.46 | 0.07 |
| **Rectal cancer** |  |  |  |  |  |  |
| **No. of cases** | 216 | 188 | 152 | 109 | 83 | 216 |
| **Total red meat** |  |  |  |  |  |  |
| **HR (95% CI)†** | 1.14 (0.92-1.41) | 1.08 (0.84-1.38) | 1.37 (1.05-1.78) | 1.40 (1.01-1.94) | 1.31 (0.94-1.84) | 1.20 (0.92-1.57) |
| ***P* for trend** | 0.24 | 0.56 | 0.02 | 0.04 | 0.11 | 0.18 |
| **Unprocessed red meat** |  |  |  |  |  |  |
| **HR (95% CI)†** | 1.22 (0.88-1.70) | 1.04 (0.70-1.55) | 1.47 (0.98-2.20) | 1.09 (0.64-1.85) | 1.28 (0.75-2.20) | 1.28 (0.84-1.95) |
| ***P* for trend** | 0.24 | 0.85 | 0.06 | 0.75 | 0.37 | 0.25 |
| **Processed red meat** |  |  |  |  |  |  |
| **HR (95% CI)†** | 1.11 (0.81-1.53) | 1.21 (0.83-1.75) | 1.47 (0.99-2.18) | 1.81 (1.16-2.81) | 1.53 (0.96-2.45) | 1.20 (0.81-1.81) |
| ***P* for trend** | 0.52 | 0.32 | 0.06 | 0.009 | 0.08 | 0.37 |

^a^  Cox proportional hazards model adjusted for age, 2-year follow-up cycle, family history of colorectal cancer, prior lower gastrointestinal endoscopy, pack-years of smoking before age 30 (0, 0-4, 4-10, >10), body mass index (in kg/m^2^; <22, 22-24, 24-25, 25-27, 27-29, 29-30, 30-32, 32-35, 35-40, or ≥40), physical activity (in metabolic equivalent-hours/week; <3, 3-9, 9-18, 18-27, or ≥27), current multivitamin use, regular aspirin or NSAID use (≥2 tablets/week), total caloric intake (quintiles), alcohol consumption (in g/d; <5, 5-10, 10-15, 15-30, or ≥30), and energy-adjusted intake of folate (quintiles), calcium (quintiles), vitamin D (quintiles) and total fiber (quintiles).
