# Supplementary material for: Processed and Unprocessed Red Meat and Risk of Colorectal Cancer: Analysis by Tumor Location and Modification by Time
Source: PLoS One. 2015 Aug 25;10(8):e0135959. doi: 10.1371/journal.pone.0135959 (PMC4549221; doi:10.1371/journal.pone.0135959)
Supplement: S5 File — (DOCX) [file pone.0135959.s005.docx]

**S5 Table. RRs and 95 % CIs associated with intake of 100 and 120 g/day of red meat and 30 and 50 g/day of processed meats by cancer sub-sites ^a^**

|  | **Total red meat** | |  | **Unprocessed red meat** | |  | **Processed meat** | |
| --- | --- | --- | --- | --- | --- | --- | --- | --- |
|  | 100g | 120g |  | 100g | 120g |  | 30g | 50g |
| **HR (95% CI) for**  **Colorectal cancer** | 1.03 (0.93-1.14) | 1.03 (0.91-1.17) |  | 1.00 (0.89-1.11) | 1.00 (0.87-1.13) |  | 1.20 (1.05-1.38) | 1.36 (1.08-1.72) |
| ***P* value** | 0.59 | |  | 0.95 | |  | 0.009 | |
| **HR (95% CI) for**  **Colon cancer** | 1.01 (0.90-1.14) | 1.02 (0.88-1.17) |  | 0.99 (0.87-1.12) | 0.98 (0.84-1.14) |  | 1.19 (1.01-1.40) | 1.33 (1.01-1.75) |
| ***P* value** | 0.81 | |  | 0.82 | |  | 0.04 | |
| **HR (95% CI) for**  **Proximal colon cancer** | 1.11 (0.94-1.30) | 1.13 (0.93-1.37) |  | 1.12 (0.94-1.32) | 1.14 (0.93-1.40) |  | 1.00 (0.78, 1.27) | 0.99 (0.67, 1.49) |
| ***P* value** | 0.22 | |  | 0.20 | |  | 0.97 | |
| **HR (95% CI) for**  **Distal colon cancer** | 0.87 (0.72, 1.05) | 0.84 (0.67, 1.06) |  | 0.79 (0.65, 0.97) | 0.75 (0.59, 0.96) |  | 1.48 (1.18, 1.85) | 1.92 (1.31, 2.80) |
| ***P* value** | 0.13 | |  | 0.02 | |  | <0.001 | |
| **HR (95% CI) for**  **Rectal cancer** | 1.12 (0.88, 1.41) | 1.14 (0.86, 1.50) |  | 1.08 (0.86,1.35) | 1.10 (0.84,1.44) |  | 1.27 (0.95,1.70) | 1.48 (0.91,2.42) |
| ***P* value** | 0.35 | |  | 0.50 | |  | 0.11 | |

^a^ Cox proportional hazards model with cumulative average total red meat, unprocessed red meat, or processed red meat intake adjusted for age, 2-year follow-up cycle, family history of colorectal cancer, prior lower gastrointestinal endoscopy, pack-years of smoking before age 30 (0, 0-4, 4-10, >10), body mass index (in kg/m^2^; <22, 22-24, 24-25, 25-27, 27-29, 29-30, 30-32, 32-35, 35-40, or ≥40), physical activity (in metabolic equivalent-hours/week; <3, 3-9, 9-18, 18-27, or ≥27), current multivitamin use, postmenopausal status and hormone use (premenopausal, and never, past and current users of postmenopausal hormone), regular aspirin or NSAID use (≥2 tablets/week), total caloric intake (quintiles), alcohol consumption (in g/d; <5, 5-10, 10-15, 15-30, or ≥30), and energy-adjusted intake of folate (quintiles), calcium (quintiles), vitamin D (quintiles) and total fiber (quintiles).
